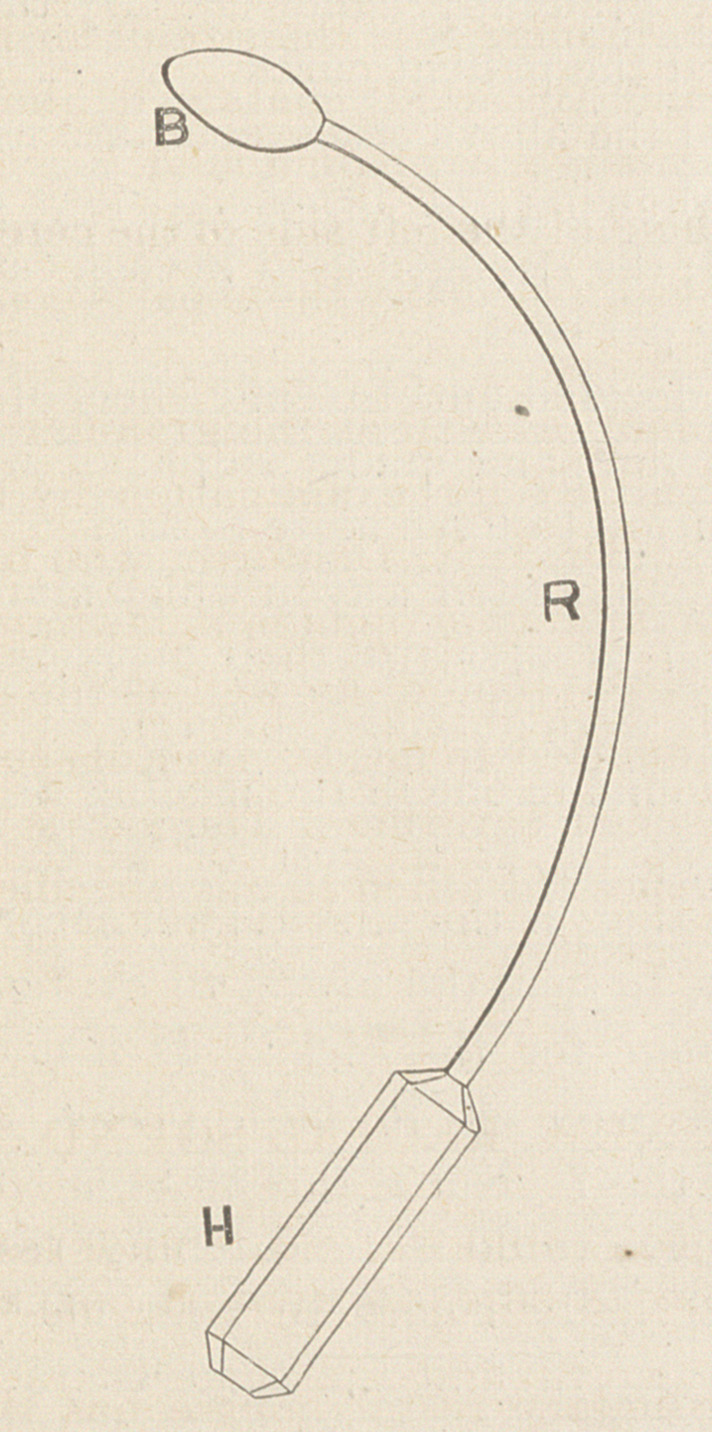# A New Rectal Sound

**Published:** 1873-11-01

**Authors:** Edmund Andrews

**Affiliations:** Professor of Surgery in Chicago Medical College; Chicago, No. 65 Randolph street


### A NEW RECTAL SOUND.

BY EDMUND ANDREWS, M.D., PROFESSOR OF SURGERY IN CHICAGO MEDICAL COLLEGE.

The ordindary rectal bougie has this absurd defect, that its form has no relation to the curve of the canal which it is intended to explore. The name *rectum* (signifying straight), seems to have been given by the ancient anatomists from dissections upon animals, and in ignorance of the fact that in man this gut is concave anteriorly to the extent of nearly one-third of a circle. The ancient tradition has not yet died out of the instrument shops, for to this day the rectal bougies are all made as straight as the rectum itself was supposed to be 2,000 years ago. The consequence is that in a healthy rectum these bougies can rarely be inserted with any safety more than five inches, and are utterly worthless for exploration beyond that distance.

In order to obtain a better form, I had a smooth bulb of hard rubber riveted upon a somewhat flexible staff of Britannia metal, which was bent approximately to the form of the rectum as it lies in the cadaver.

By repeated trials upon living patients, and carefully modifying the curve of the staff, I was able at length to arrive at what seems to be the best form. The resulting instrument is here figued on a scale one-quarter the actual size. H is a handle of ebony. R is a rod of soft steel, German silver, or Britannia. This rod projects thirteen inches from the handle, and is bent at a curve which increases as you go towards the tip: that is, say, near the handle the curve has a radius of $8\frac{1}{2}$ inches, while near the tip the radius is only 5 inches. The end of the rod terminates in a screw which enters the hard rubber bulb, B. Half a dozen of these bulbs should be made of different sizes, so that by simply changing them a sound is obtained fitted to a stricture of any calibre, This instrument will often pass in ten or twelve inches without obstruction, and can be made to press on the abdominal walls from within, so as to be felt by the surgeon's hand within two inches of the umbilicus, which is an extent of exploration which no straight instrument can ever approach.

Chicago, No. 65 Randolph street.

## Figures and Tables

**Figure f1:**